# Effect of Dietary and Age Changes on Ruminal Microbial Diversity in Holstein Calves

**DOI:** 10.3390/microorganisms12010012

**Published:** 2023-12-20

**Authors:** Yinglian Wu, Chong Jiao, Qiyu Diao, Yan Tu

**Affiliations:** Key Laboratory for Dairy Cow Nutrition, Institute of Feed Research of Chinese Academy of Agricultural Sciences, Beijing 100081, China; wyl996996@163.com (Y.W.); jiaochong2017@163.com (C.J.); diaoqiyu@caas.cn (Q.D.)

**Keywords:** calves, age, rumen bacterial, rumen fungal, volatile fatty acids

## Abstract

Ruminal microorganisms play a crucial role in the energy supply of ruminants and animal performance. We analyzed the variations in rumen bacteria and fungi at 45 d, 75 d, and 105 d by using 16SrRNA and ITS sequencing data and investigated their correlation with rumen fermentation. According to the results, rumen microflora tended to gradually mature with age, and bacterial and fungal establishment gradually stabilized. Upon comparing the three periods, the concentration of propionic acid increased significantly (*p* < 0.05) after weaning, and weaning accompanied by a transition in diet remarkably decreased (*p* < 0.05) rumen diversity in the short term and induced a corresponding change in the rumen microbiota composition. *Bacteroidota*, *Actinobacteriota*, and *Firmicutes* were the core bacterial phyla for all age periods. *Ruminococcus*, *NK4A214_group*, *Sharpea*, *Rikenellaceae_RC9_gut_group*, and *norank_f__Butyricicoccaceae* were the markedly abundant bacterial genera in pre-weaning. After weaning, the relative abundance of *Erysipelotrichaceae_ UCG-002, Eubacterium_ruminantium_group*, and *Solobacterium* significantly increased (*p* < 0.05). The relative abundance of *Acetitomaculum* increased with age with the greatest abundance noted at 105 d (37%). The dominant fungal phyla were *Ascomycota* and *Basidiomycota*, and *Aspergillus* and *Xeromyces* were the most abundant fungal genera after weaning. *Trichomonascus*, *Phialosimplex*, and *Talaromyces* were enriched at 105 d. However, the low abundance of *Neocallimastigomycota* was not detected throughout the study, which is worthy of further investigation. In addition, correlations were observed between age-related abundances of specific genera and microbiota functions and rumen fermentation-related parameters. This study revealed that rumen microbiota and rumen fermentation capacity are correlated, which contributed to a better understanding of the effects of age and diet on rumen microbiology and fermentation in calves.

## 1. Introduction

The rumen is a special organ in ruminants and has a complex microbial community composed of bacteria, fungi, and protozoa. Cellulosic materials in the feed are degraded into available nutrients (e.g., volatile fatty acids (VFAs)) through fermentation, thereby providing energy for the growth of the host and leading to the production of milk and meat products for human consumption [1,2]. Rumen microbes and the productive performance of ruminant hosts are closely associated. For example, rumen bacterial taxa are associated with milk production and composition in dairy cows [3,4]. Meanwhile, different microbial interaction models were found among animals with different feed efficiencies [5]. In addition, the abundance of specific bacteria can help predict the growth performance of beef cattle [6]. The rumen microbiota is also fundamental to processes such as digestive tract development, nutrient supply, and immune system establishment [7,8,9], which are essential for ruminants. While many studies have investigated rumen microorganisms, basic questions about their evolving regularity have remained unanswered. The composition of these complex microorganisms and how they evolve during an animal’s lifetime remain unelucidated.

The development and structure of rumen microbiota are related to the ruminant’s age. A multi-omics analysis revealed that microbial colonization of the fetal gut begins in utero [10]. Guzman et al. [11] found typical functional microbial periods such as methanogenic fiber-degrading bacteria and morphogenic bacteria in the calf rumen within 20 min after birth. In their intensive studies on rumen microorganisms in calves from birth to adulthood, Jami et al. and Koringa et al. observed a rapid decrease in aerobic and facultative anaerobic genera in the calf rumen at 3 d after birth, whereas there was a rapid increase in anaerobic genera [12,13]. Furman et al. also noted a significant prioritization effect for species observed in the early growth period in the rumen. Therefore, the dynamics of taxa of late evolutionary generations depend on the microbial composition during early life [14]. Saro et al. revealed that a prolonged intervention in early life affects the microbial community composition of rumen and persists for several weeks after the intervention [15]. The effect of age on microbial communities in ruminants, such as buffalo [13], cows [16], and lambs [17,18], has been reported. Studies have reported age-related changes in the rumen microbiota [12,19]. Moreover, age-related microbial changes are closely associated with inflammation [20] and methane emission [21]. It was reported that rumen bacterial composition changed with dietary nutrient levels, and the different roughage sources had an effect on the rumen microbiota [22,23]. Lin et al. found that dietary changes selectively altered the metabolic cascade of specific gastrointestinal microbiomes, and the transition from forage-dominated to grain-dominated changed specific populations of microorganisms in favor of starch degradation, primarily by narrowing the hydrolysis rate of plant biomass [24]. However, as calves gradually grow older, their dietary structure must be adjusted to satisfy their nutritional requirements at different life stages. Specific information on the integrated effects of diet and age on the rumen microbial composition and function in calves is lacking, which limits the accurate feeding management of ruminants. Information on these effects must be gathered.

Therefore, this study investigated rumen fermentation parameters and bacterial and fungal diversity in cattle of different ages before and after weaning by using 16SrRNA and ITS sequencing methods. The goal was to determine differences in the composition of rumen bacteria and fungi in cattle fed with specific rations. This information will improve our understanding of rumen microbial ecology, eventually providing a theoretical basis for precision feeding and management of ruminants.

## 2. Materials and Methods

### 2.1. Experimental Animals and Management

The trial was conducted in September 2017 at a ranch for Shandong Yinxiang Weiye Group Co., Ltd., Heze, China, (N 34°85′, E 115°45′). The experimental protocol was approved by the Society for Animal Ethics and Humane Animal Protection, Chinese Academy of Agricultural Sciences (protocol# FRI-CAAS-20200918).

Thirty Holstein newborn healthy female calves with no antibiotic use in the month prior to sampling were selected, and the calves had similar body weights (BW: 33 ± 2 kg) and age (7 ± 3 d). The separation of calves from their mothers was immediately after birth. They were fed enough colostrum on time after birth. The calves were ear-tagged and their numbers were recorded, after which they were reared in individual pens (2 m × 1.5 m).

The calves were fed pasteurized milk twice a day (06:30 a.m. and 05:00 p.m.) at 4 L/day from 7 to 14 d and 6 L/day from 14 to 60 d, after which they were weaned for 1 week from 60 d (67 d). During this period, the amount of milk fed was reduced by 15% on the first day of the weaning transition, 30% on the second day, 45% on the third day, and gradually reduced until they were completely weaned at 67 d. From the beginning of 7 d of age, the animals were fed with a starter (Table S1). The starter was fed twice a day (06:30 a.m. and 05:00 p.m.), and the amount of starter feed was added according to the previous diet, ensuring that there was a small amount of leftover feed in the trough. These animals were fed oat hay once daily from 30 d of age, 80 g/day from 30 to 60 d, 200 g/day from 60 to 90 d, and 300 g/day from 90 to 120 d. Table 1 presents the primary nutrient levels of the different diets. The amount of nutrients provided by the diet meets the nutritional requirements of the animal at each stage of development [25]. The pretrial was conducted for 7 d, and the entire experiment was conducted for 120 d. The BW of each calf was measured before morning feeding at 14, 30, 60, 90, and 120 d of age. During the whole experimental period, the calves had free access to water. The sheds were cleaned, disinfected with a disinfectant (2–4%NaOH), and replaced with new paddy husk before the experiment. Disease prevention and treatment of calves was ensured according to the normal management procedures of the farm.

### 2.2. Sample Collection

**Figure 1:**
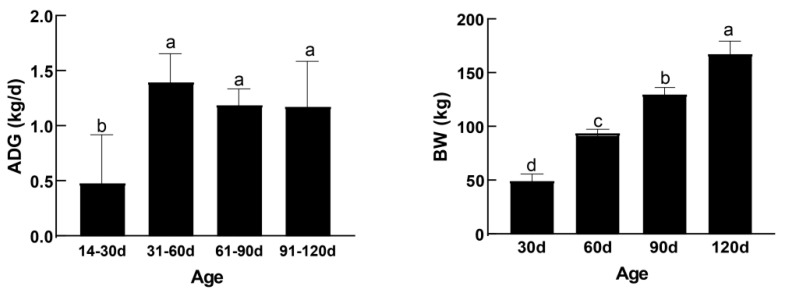
Average daily gain and body weight of calves at different periods. Different letters represent significant differences, *p* < 0.05; same letters represent insignificant differences, *p* > 0.05.

After the formal trial began, eight calves with good health and similar average daily gain (ADG) (1.19 ± 0.12 kg) throughout the trial period were selected. Their changes in ADG and BW are shown in Figure 1. Table 2 presents the type of diets, total dry matter (DMI), and the amount of major nutrients taken by the calves at different age periods. The total feed intake of the calves increased significantly with age. The intake of crude protein and dietary fiber increased significantly after weaning. Using an oral rumen tube, rumen fluid was collected 2 h after morning feeding at 45, 75, and 105 d. Three of the calves were eliminated because of disease at 105 d. Therefore, these three cows were removed from further analyses. A part of the collected rumen fluid was stored at −80 °C for microbial sequencing analysis, and it is noteworthy that this study focused on the planktonic community. The other part was stored at −20 °C for determining VFAs and ammonia nitrogen (NH_3_-N).

### 2.3. Measurement of Fermentation Parameters

The collected rumen fluid was thawed and centrifuged to extract the supernatant for analysis. Immediately after sampling the rumen fluid, the pH of the rumen fluid was measured using a portable pH meter (8362sc Portable pH meter; Biyuntian biotechnology Co., Ltd., Shanghai, China). The VFA concentration was determined through gas chromatography, and the NH_3_-N concentration was measured using the colorimetric method with hypochlorite phenol [26].

### 2.4. DNA Extraction and 16SrRNA Sequencing

After the sample was thawed, total microbial genomic DNA was extracted from rumen fluid samples according to the instructions of the FastDNA^®^ Spin Kit for Soil (MP Biomedicals, Solon, GA, USA). After DNA extraction was completed, the DNA quality and concentration were determined through 1.0% agarose gel electrophoresis using the NanoDrop^®^ ND-2000 Spectrophotometer (Thermo Scientific Inc., Waltham, MA, USA) and stored at −80 °C. The extracted DNA was used as a template for amplifying bacterial 16S rRNA by using primers [27], 338F (5′-ACTCCTACGGGAGGCAGCAG-3′) and 806R (5′-GGACTACHVGGGTWTCTAAT-3′), and an ABI GeneAmp^®^ 9700 PCR thermal cycler (ABI, Vernon, CA, USA) in the V3–V4 of the highly variable region of the gene. The ITS region of the fungal genomic DNA was amplified using primers [28], ITS1F (5′-CTTGGTCATTTAGAGGAAGTAA-3′) and ITS2R (5′-GCTGCGTTCTTCATCGATGC-3′). The amplification procedures followed were according to Ma et al. [29]: initial denaturation at 95 °C for 3 min, followed by 27 cycles of denaturation at 95 °C for 30 s, annealing at 55 °C for 30 s, extension at 72 °C for 45 s, single extension at 72 °C for 10 min, and final extension at 72 °C for 10 min. PCR products were recovered from the 2% agarose gels, purified using the QIAquick Gel Extraction Kit (Qiagen, Hilden, Germany), detected through 2% agarose gel electrophoresis, and quantified using a Quantus™ Fluorometer (Promega, Madison, WI, USA). The purified PCR products were used for constructing libraries by using the NEXTFLEX Rapid DNA-Seq Kit. High-throughput sequencing(2 × 300 bp) was performed by Shanghai Meiji Biomedical Technology Company Limited (Shanghai, China) using the Illumina Miseq sequencing platform (Illumina Corporation, San Diego, CA, USA). The raw data were uploaded to the NCBI SRA database (BioProject ID:PRJNA1047267).

### 2.5. Bioinformatics Analysis

Rumen fluid samples were quantified using the fastp [30] (https://github.com/OpenGene/fastp, version 0.19.6, accessed on 30 May 2022) software for raw sequences and the FLASH [31] (http://www.cbcb.umd.edu/software/flash, version 1.2.11, accessed on 30 May 2022) software for splicing. The sequences obtained after quality control splicing were clustered, and chimeras were removed based on 97% similarity using the UPARSE [32,33] software (http://drive5.com/uparse/, version 7.1, accessed on 30 October 2022) with an operational taxonomic unit (OTU). To reduce the impact of sequencing depth on subsequent data analyses of alpha diversity and beta diversity as much as possible, all sample sequence numbers were normalized. Bacteria were annotated using the RDP classifier [34] (http://rdp.cme.msu.edu/, version 2.11, accessed on 10 November 2022) when compared to the Silva 16S rRNA gene database (v138) for OTU species taxonomy with a confidence threshold of 70%. Fungi were annotated by comparison to the Unite (Release 8.0 http://unite.ut.ee/index.php, accessed on 30 December 2022) part of the fungal database. The community composition of each sample was determined at different species taxonomic levels. To explore the functions performed by the rumen microbial community in vivo, we performed a 16S functional prediction analysis by using the PICRUSt2 [35] (version 2.2.0) software.

### 2.6. Statistical Analysis

Rumen pH and fermentation parameters were analyzed using a one-way ANOVA and the Tukey by SAS (version 9.4, SAS Institute Inc., Cary, NC, USA) software. All microbiological data analyses were performed on the online Majorbio Cloud Platform (https://cloud.majorbio.com). The alpha diversity index was calculated using the mothur [36] software (https://www.mothur.org/wiki/Download_mothur, version 1.30.2, accessed on 20 October 2022), and differences in alpha diversity between periods were further analyzed using the Wilcoxon rank-sum test. Principal coordinate analysis (PCoA) with analysis of similarities (ANOSIM) (999 permutations) was performed on beta diversity to test for similarities or differences between periods through comparative analysis of microbial diversity between these periods. The microbial taxa with significant differences in abundance from the phylum level to the genus level among the three age periods were comparatively analyzed using linear discriminant analysis effect size (LEfSe) [37]. The relationships between fermentation parameters and microorganisms were analyzed at the genus level, and the results are displayed visually in a heat map.

## 3. Results

### 3.1. Fermentation Parameters

**Figure 2:**
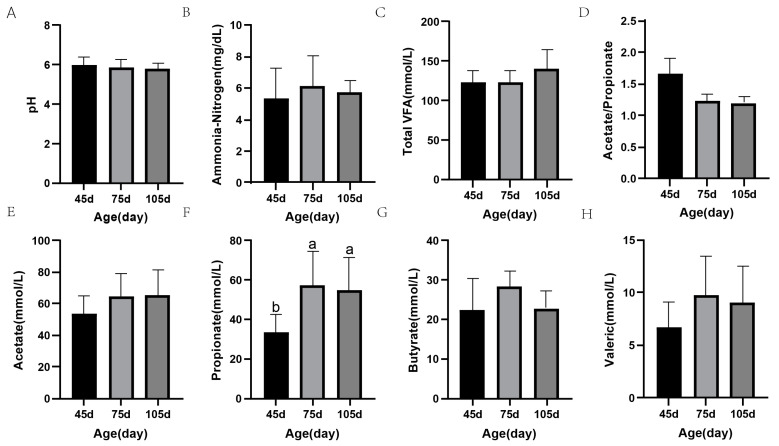
Fermentation parameters of rumen fluid. Different letters represent significant differences, *p* < 0.05. (**A**) pH. (**B**) Ammonium Nitrogen. (**C**) Total VFA. (**D**) Acetate/Propionate. (**E**) Acetate. (**F**) Propionate. (**G**) Butyrate. (**H**) Valerate. Same letters represent insignificant differences, *p* > 0.05.

Figure 2 presents the rumen fermentation characteristics of the experimented-upon animals. No significant differences in rumen pH, NH_3_-N, TVFA, acetate/propionate, acetate, butyrate, and valeric were observed between the pre-weaning and post-weaning periods (*p* > 0.05) (Figure 2A–E,G,H). However, the concentration of total VFA in the rumen of calves increased, whereas that of acetate/propionate gradually decreased in trend with age. The propionate concentration was significantly higher after weaning than after pre-weaning (*p* < 0.05) (Figure 2F). Compared to those at 75 d, the rumen fermentation parameters exhibited no age-dependent variations at 105 d (*p* > 0.05).

### 3.2. Characteristics of Rumen Microbial Diversity

#### 3.2.1. Bacterial Diversity and Richness

**Figure 3:**
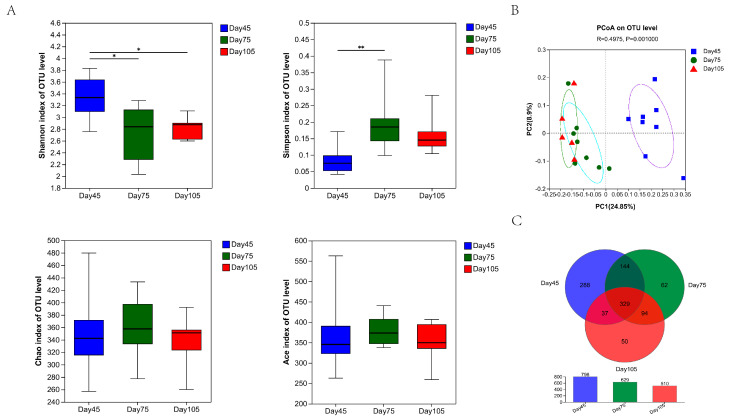
Variation of diversity in rumen bacterial communities in calves at 45 d, 75 d, and 105 d and OTUs. (**A**) Alpha diversity index. * denotes significant differences between groups (*p* < 0.05), and ** denotes significant difference between groups (*p* < 0.01). (**B**) Principal coordinate analysis of rumen bacterial communities at the OUT level. (**C**) Venn diagram of OTUs.

To explore the variation of rumen microbial diversity and richness in calves at different ages, we calculated the alpha diversity. According to the results obtained, some amount of variation was noted in the rumen bacterial diversity at different calf ages. The Shannon index was significantly reduced (*p* < 0.05) between the pre-weaning and post-weaning periods. The differences in the Chao and Ace indices were not statistically significant, but the Simpson index significantly increased (*p* < 0.001) at 75 d. In contrast, no significant differences in the Shannon, Simpson, Chao, and Ace indices were observed between 75 d and 105 d (Figure 3A).

Meanwhile, a PCoA plot was used to analyze the similarity or difference in bacterial communities between different age periods, in which each point represents a sample and the points with the same color are from the same group. The closer the distance between two points, the smaller the difference between the two communities. As shown in Figure 3B, principal coordinate 1 accounted for 24.85% of the total variation, and principal coordinate 2 accounted for 8.9% of the total variation. The bacterial community composition at 45 d was obviously different from those at 75 and 105 d, which indicates differences in the rumen bacterial composition of calves at different ages. Furthermore, the Venn diagram illustrates this at the OTU level (Figure 3C). The findings at 45 d, 75 d, and 10 d shared 329 core OTUs, and 288, 62, and 50 OTUs were unique, respectively. The results indicate that the number of unique OTUs gradually lowered with an increase in age. Each age had its own unique bacterial community, and at a later period, the rumen bacterial communities became more similar.

#### 3.2.2. Fungal Microbial Diversity and Richness

**Figure 4:**
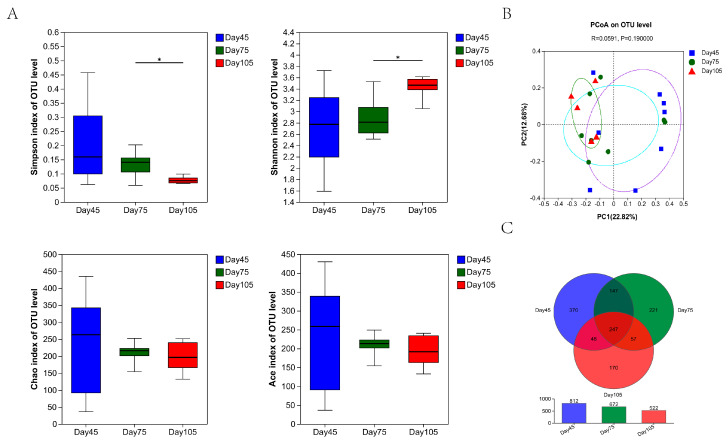
Variation of diversity of rumen fungal communities in calves at 45 d, 75 d, and 105 d and OTUs. (**A**) Alpha diversity index. * denotes significant differences between groups (*p* < 0.05). (**B**) Principal coordinate analysis of rumen fungal communities at the OUT level. (**C**) Venn diagram of OTUs.

The alpha diversity results of rumen fungi revealed that the Shannon index was significantly higher (*p* < 0.05) whereas the Simpson index was significantly lower (*p* < 0.05) at 75 d than at 105 d. This indicates a difference in rumen fungal diversity between 75 and 105 d. However, the Chao and Ace indices exhibited no significant differences among the three periods (Figure 4A). Hence, weaning had an effect on diversity but no significant effect on richness.

At the OTU level, a PCoA (Figure 4B) revealed that primary coordinate 1 accounted for 22.82% of the total variation, and primary coordinate 2 accounted for 12.68% of the total variation. The fungal region at 105 d exhibited significant clustering. As shown in the Venn diagram (Figure 4C), the three periods shared 247 core OTUs and had 370, 221, and 170 unique OTUs distributed in the sequence, respectively. In general, differences were noted in the rumen fungal compositions among the three age periods.

### 3.3. Ruminal Microbial Composition and Differences

#### 3.3.1. Bacterial Composition and Differences

**Figure 5:**
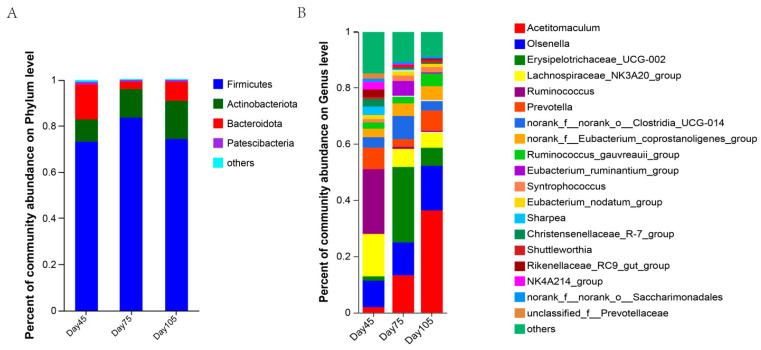
The composition of rumen bacteria at phylum and genus levels at 45 d, 75 d, and 105 d. (**A**) The composition of rumen bacteria at the phylum level; (**B**) The composition of rumen bacteria at the genus level.

In total, 684,831 bacterial valid sequences were obtained from 21 samples by using 16SrRNA gene sequencing to investigate changes in the rumen microbial community in this study. At the phylum level, 18 bacterial phyla were identified, with *Firmicutes*, *Actinobacteriota*, and *Bacteroidota* being the dominant phyla. The relative abundance of rumen *Firmicutes*, *Actinobacteriota*, *Bacteroidota*, and *Patescibacteria* in the pre-weaning period of calves was 73.17%, 9.78%, 14.94%, and 1.24% (Figure 5A). In contrast, the relative abundance of *Firmicutes* and *Actinobacteriota* increased significantly in the post-weaning period, whereas that of *Bacteroidota* decreased significantly. The relative abundance of *Firmicutes* decreased significantly at 105 d, whereas that of *Actinobacteriota* and *Bacteroidota* increased.

At the genus level, 316 bacterial genera were identified, and 9 bacterial genera exhibited an abundance of >2% in the resulting sequences. The abundance of the dominant bacterial genera, *Acetitomaculum*, *Olsenella*, and *norank_f_Eubacterium_coprostanoligenes_group*, gradually increased with age, whereas that of *Lachnospiraceae_NK3A20_group* and *Ruminococcus* gradually decreased. Compared to that at 45 d and 105 d, the abundance of *Erysipelotrichaceae_UCG-002*, *Prevotella*, *norank_f_norank_o_Clostridia_UCG014*, and *Eubacterium_UCG014* increased in the rumen at 75 d. *Eucbacterium_ruminantium_group* had the highest relative abundance, and *Ruminococcus_gauvreauii_group* had the highest abundance at 105 d (Figure 5B).

**Figure 6:**
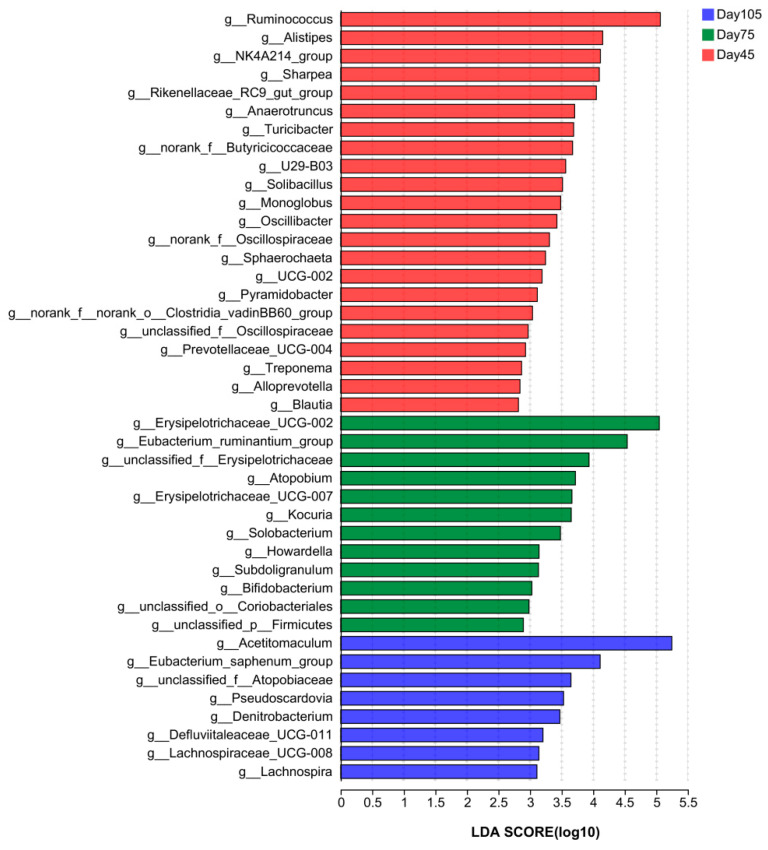
Linear discriminant analysis effect size analysis of bacterial taxa within the three periods. Histogram of linear discriminant analysis (LDA) scores at the genus level, with the length of the bars representing LDA scores. Significant differences are defined as *p* < 0.05 and scores > 2.5 for LDA.

To identify the characteristics of differences in variation of bacterial abundance between different age periods, a linear discriminant analysis (LDA) was performed to characterize flora at the genus level (Figure 6). The results showed *Ruminococcus*, *Alistipes*, *NK4A214_group*, *Sharpea*, and *Rikenellaceae_RC9_gut_group* as being remarkably enriched before weaning (LDA > 2.5, *p* < 0.05), and the relative abundance of *Erysipelotrichaceae_UCG-002*, *Eubacterium_ruminantium_group*, and *Solobacterium* as significantly increased after weaning (LDA > 2.5, *p* < 0.05). However, *Acetitomaculum* was identified as the characteristic flora at 105 d (LDA > 2.5, *p* < 0.05).

#### 3.3.2. Fungal Composition and Differences

**Figure 7:**
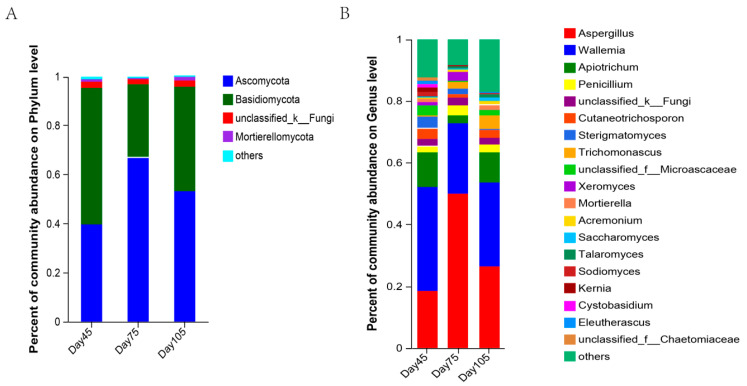
The composition of rumen fungi at the phylum and genus levels at 45 d, 75 d, and 105 d. (**A**) The composition of rumen fungi at the phylum level; (**B**) The composition of rumen fungi at the genus level.

At the phylum level, 13 fungal phyla were identified in all the samples, and the highest abundance was noted in *Ascomycota* and *Basidiomycota* at the three stages, which accounts for more than 90% of the total abundance. The relative abundance of *Ascomycota* was 37.66% during pre-weaning, and its abundance increased after weaning, subsequently reducing to 52.80% at 105 d. Simultaneously, the abundance of *Basidiomycota* decreased from that at 45 d by 27.82% at 75 d and decreased by 14.42% at 105 d (Figure 7A).

At the genus level, 394 fungal genera were identified. At 45, 75, and 105 d, *Aspergillus, Wallemia*, and *Apiotrichum* were the dominant genera (Figure 7B). In the pre-weaning period, the relative abundance of *Aspergillus*, *Wallemia*, and *Apiotrichum* was higher; the abundance of *Aspergillus* significantly increased after weaning, while the relative abundance of *Wallemia*, *Apiotrichum*, and *Cutaneotrichosporon* decreased significantly. At 105 d, the relative abundance of *Aspergillus*, *Wallemia*, and *Apiotrichum* increased.

**Figure 8:**
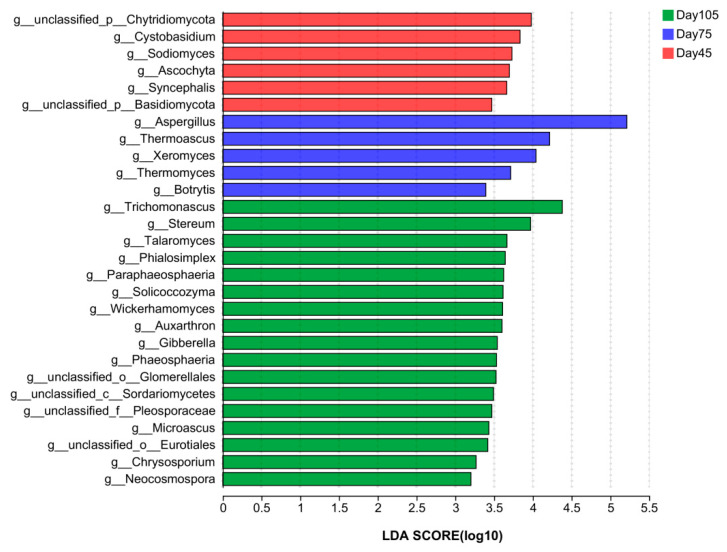
Linear discriminant analysis effect size analysis of bacterial taxa within the three periods. A histogram of LDA scores at the genus level, with the length of the bars representing LDA scores. Significant differences are defined as *p* < 0.05 and scores > 2.5 for LDA.

Figure 8 presents the comparative analysis of differences in the abundance of rumen fungi. At the genus level, the characteristics of *Cystobasidium* and *Sodiomyces* were enriched in the pre-weaning period. *Aspergillus*, *Thermoascus*, and *Xeromyces* were identified as characteristic post-weaning flora with LDA scores of >4. The characteristics of *Trichomonascus*, *Phialosimplex Talaromyces*, *Wickerhamomyces*, *unclassified_c__Sordariomycetes*, and *Microascus* were significantly enriched at 105 d (LDA > 2.5, *p* < 0.05; Figure 8).

### 3.4. Function Prediction

**Figure 9:**
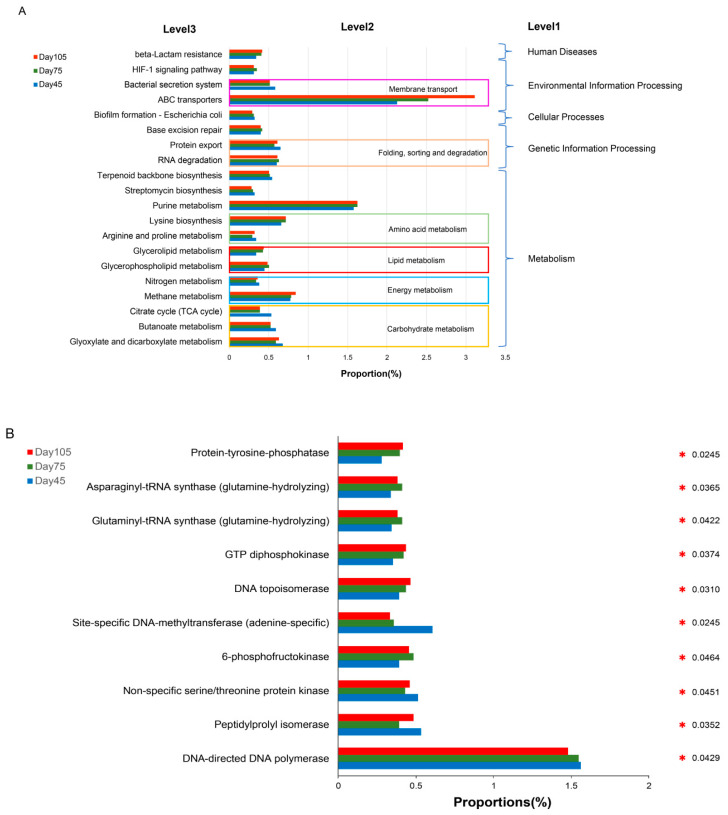

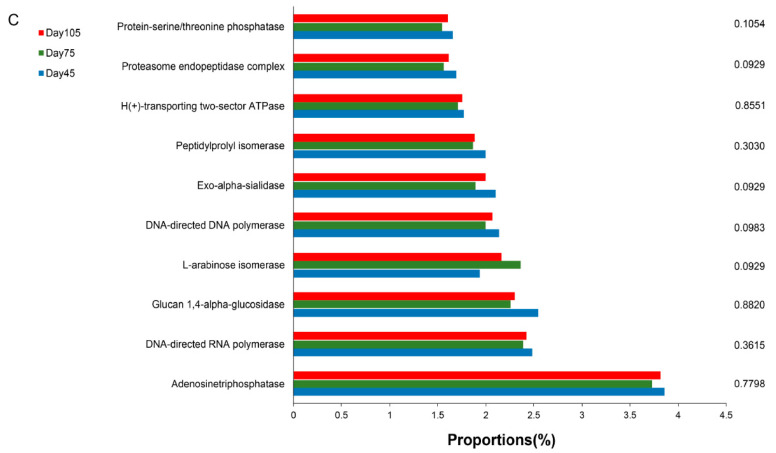
PlCRUSt functional prediction analysis: (**A**) the top 20 KEGG metabolic pathways at the three age periods of bacteria with significant differences (*p* < 0.05); (**B**) the percentage of enzyme functional class abundance in the top 10 bacteria with significant differences (*p* < 0.05); and (**C**) the percentage of enzyme functional class abundance in the top 10 fungi.

The analysis of functional prediction of bacterial communities using PlCRUSt was compared with data from the Greengene database to obtain the corresponding KO functional information and its abundance. Figure 9A presents the top 20 KEGG metabolic pathways with significant differences (*p* < 0.05) in abundance at three age periods. The level 1 metabolic pathway indicates that genes were mainly concentrated in metabolism, genetic information processing, environmental information processing, cellular processes, and human diseases. The level 2 metabolic pathway demonstrates that most genes were concentrated in amino acid metabolism, carbohydrate metabolism, energy metabolism, lipid metabolism, and membrane transport pathways. Most genes in level 3 metabolic pathways were closely related to biological processes, such as amino acid anabolism and energy metabolism. The data showed significantly higher gene categories of carbohydrate metabolism, CAZymes, associated with polysaccharide degradation in the pre-weaning period than in the post-weaning period. Information on the top 10 enzymes with significant differences in abundance (*p* < 0.05), obtained from the KEGG database, were consistent with changes in KEGG functional categories. When the abundance of functional classes of related enzymes in the three age periods of fungi was compared, most genes were found to be associated with carbohydrate metabolism versus protein metabolism but exhibited no significant difference (*p* > 0.05) (Figure 9B,C).

### 3.5. Correlation Analysis

#### 3.5.1. Correlations among the Microbiota

**Figure 10:**
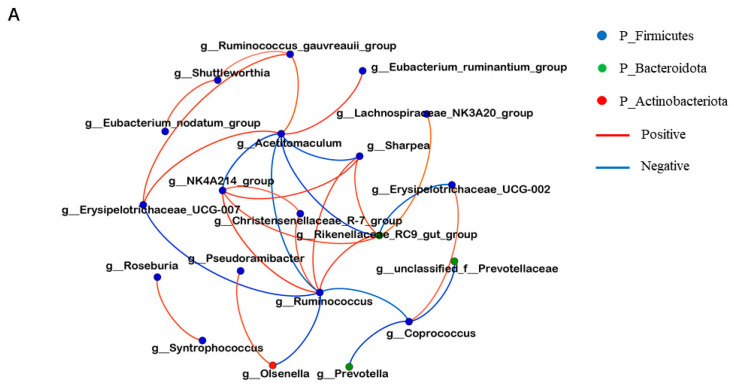

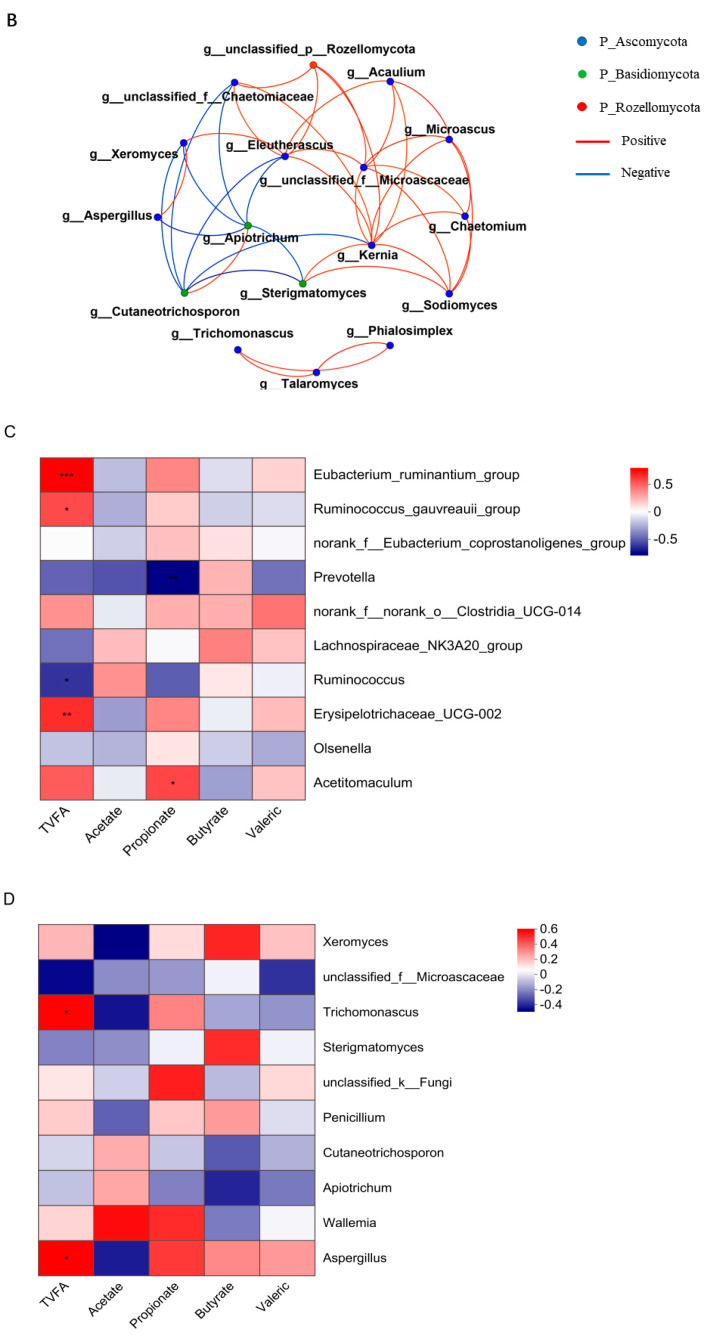
Correlation network analysis plots constructed for the top 20 microbiota in terms of relative abundance with R > 0.5, *p* < 0.05: (**A**) interactions among bacteria; (**B**) interactions among fungi. Spearman correlation analysis of microbiota and VFA: (**C**) correlation analysis of bacteria and VFA; (**D**) correlation analysis of fungi and VFA. Colors represent the correlation coefficients, with red representing a positive correlation and blue denoting a negative correlation. R > 0.5,* *p* ≤ 0.05, ** *p* ≤ 0.01, *** *p* ≤ 0.001.

From the top 20 bacterial and fungal genera in terms of relative abundance, correlation network analysis plots were constructed using the results of a Spearman correlation analysis and selecting bacteria with R > 0.5 and *p* < 0.05. *Acetitomaculum*, *NK4A214_group*, *Ruminococcus*, and *Rikenellaceae_RC9_gut_group* play crucial roles in the network with a higher number of interactions. For instance, at the bacterial genera level, significant negative correlations were noted between *Ruminococcus* and *Acetitomaculum*, whereas significant positive correlations were observed between *NK4A214_group* and *Rikenellaceae_RC9_gut_group* (Figure 10A). At the fungal genera level, significant positive correlations were noted between *Aspergillus* and *Xeromyces*, whereas significant negative correlations were observed between *Talaromyces* and *Trichomonascus* (Figure 10B).

#### 3.5.2. Microbiota and Fermentation Parameters

As shown by the heat map (Figure 10C), some ruminal bacteria (genus level) were remarkably correlated with ruminal fermentation parameters. For example, *Acetitomaculum*, *Ruminococcus_gauvreauii_group*, *Erysipelotrichaceae_UCG-002*, *Ruminococcus gauvreauii group*, and *Eubacterium_ruminantium_group* were significantly positively related to TVFA (*p* < 0.05). In contrast, *Ruminococcus* was significantly negatively correlated with TVFA (*p* < 0.05; R = −0.53). *Acetitomaculum* was significantly positively correlated with propionate (*p* < 0.05; R = 0.55), whereas *Prevotella* was significantly negatively correlated with propionate (*p* ≤ 0.01; R = −0.69). At the fungal genera level (Figure 10D), *Aspergillus* and *Trichomonascus* demonstrated significant positive correlations with TVFA (*p* < 0.05; R = 0.51, R = 0.50).

## 4. Discussion

The rumen is a complicated ecosystem comprising diverse microorganisms. Ruminants depend on these microbes to degrade ingested nutrients through fermentation and provide the majority of energy for life activities. More importantly, in young ruminants, rumen maturation and its microbiota evolution are directly related to the host animal’s health and production performance [38,39]. Age and diet are dominant factors influencing rumen microbiota in hosts, Hence, identifying their effects on the microbial community is significant for further designing optimal management strategies.

Rumen fermentation parameters are critical indicators of the rumen fermentation condition and developmental function and are mainly mediated by different rumen microorganisms. Volatile fatty acids (VFAs) are produced during the fermentation process of feedstuffs. VFAs are essential for rumen development, performance, and body metabolism and fulfil more than 70% of the energy requirements of ruminants [40,41]. This study results revealed that rumen concentrations of acetate, propionate, butyrate, valerate, and TVFA were higher whereas that of A/P was lower after weaning. This reflects the greater fermentation capacity of the rumen microbiota in the post-weaning period, and indicates that the rumen of post-weaning calves can produce more VFA for host utilization. These results are consistent with those of a previous study [42]. Of course, rumen VFA concentrations are also closely related to feed intake. The total DMI of calves gradually increased at different stages (Table 2). Rumen TVFA concentrations have been found to increase with increases in solid feed intake [26], which is similar to our findings. It is noteworthy that the propionate concentration was remarkably higher at 75 d than at 45 d and decreased at 105 d. This may be due to adaptation to a change in diet after weaning and the relatively high intake of starch and CP in the starter diet, which increased the propionate concentration, and the later increased intake of fiber significantly increased the acetate concentration in the rumen. In addition, the TVFA concentration increased gradually with age, and the lower pH of the rumen after weaning also indicated this increase. This may be due to VFA accumulation in the rumen caused by the imbalance between VFA production and rumen epithelial absorption. NH_3_-N in the rumen is produced through degradation of protein or non-protein nitrogenous substances in the feed [43]. It is a precursor substance for MCP synthesis in the rumen and can serve as a nitrogen source for the growth of rumen microbes. However, the specific mechanisms underlying these functions of NH_3_-N need to be further explored.

The microbial diversity index is a common index used for evaluating ecosystem stability. According to some studies [44,45], the rumen microbial diversity in cattle and sheep changes significantly with age, which is consistent with our study results. A remarkable variation in the Shannon and Simpson indices of bacteria was observed after weaning, which indicates a significant decline in bacterial diversity. This may be due to the weaning transition period, wherein switching of diet types caused weaning stress in calves, and the rumen microbiota failed to immediately adapt to drastic dietary changes, thereby leading to a decrease in bacterial diversity. This variation was similar to the change in bacterial diversity observed after the weaning of lambs [46]. In addition, no significant difference in bacterial diversity was noted between 75 and 105 d. Beta diversity further illustrated this issue as well. However, the Venn diagram showed that differences were still present between these two age periods. Here, we noted that fungal diversity significantly increased at 105 d compared with 75 d, which may be related to the increased crude fiber intake during the later period (Table 2). Furthermore, the Venn diagram results for bacteria and fungi demonstrated that different age periods resulted in a unique decrease in the number of OTUs. This also proved that, with an increase in age, rumen development tends to gradually mature and rumen microbial establishment gradually stabilizes and that these rumen microbiota cluster toward more similar microbiota.

The microbial composition and function in the rumen vary at different life stages of cattle. *Firmicutes* and *Bacteroides* are the dominant bacteria in the rumen of dairy cows [47,48]. In this study, at the bacterial phylum level, the core microbiota consisted of *Bacteroides*, *Actinobacteria*, and *Firmicutes*. These were the three dominant phyla. The relative abundance of *Firmicutes* in the rumen significantly increases after weaning, whereas that of *Bacteroides*, which is crucial for milk digestion, decreases after the feeding of solid food [49]. This is consistent with the changes in bacterial abundance observed after the weaning of lambs [45]. The increased relative abundance of *Firmicutes* is associated with solid feed intake, energy acquisition, and feed efficiency [50,51]. On observing the three age periods differing at the bacterial genus level, we noted that the relative abundance of *Ruminococcus*, *NK4A214_group*, *Sharpea*, *Rikenellaceae_RC9_gut_group*, and *norank_f__Butyricicoccaceae* was higher at 45 d. *NK4A214* is a Gram-negative bacterium in Firmicutes that can utilize or degrade fibers and polysaccharides from the diet that are not easily degraded [52]. Studies have reported higher abundance of *NK4A214_group* in the rumen of concentrated feed-fed yaks. *NK4A214_group* belongs to the *Ruminococcus* genus, and such variation in its abundance may be related to its ability to digest resistant starch [53]—the content of which was higher because of the higher concentrated percentage of feeds. The relative abundance of *NK4A214_group* is positively correlated with host feed utilization and VFA metabolism [54]. *Ruminococcus* is among the most efficient bacterial genera for carbohydrate catabolism. Some of the *Ruminococcus* bacteria get their nutrients by degrading the cellulose in the host’s digestive system. *Sharpea* belongs to the *Firmicutes* phylum and can digest various carbohydrates to produce VFA [55]. The relative abundance of *Sharpea* increases as the solid intake increases after weaning, resulting in an increased ability to digest carbohydrates in early weaned lambs [46]. In the present experiment, the relative abundance of *NK4A214_group*, *Ruminococcus*, and *Sharpea* was lower after weaning, possibly because of the interactions among the microbiota. The Spearman correlation analysis revealed that these three bacteria are negatively correlated with *Acetitomaculum*. In the rumen of buffalo, the relative abundance of *Ruminococcus sp*. decreases with the age of the animal, which suggests the additional roles of other microorganisms or ecological niches in carbohydrate and cellulose degradation [13]. The *Butyricoccaceae* [56] and *Rikenellaceae* families [57] are involved in carbohydrate degradation. The relative abundance of *Rikenellaceae_RC9* [48] decreases in cows fed with high-starch diets containing grease. In a study on Tan sheep, *Rikenellaceae_RC9_gut_group* exhibited a significant positive correlation with meat fat, and this bacteria may regulate fat deposition in meat by affecting VFA concentrations [58]. These experimental results demonstrate that the relative abundance of *Erysipelotrichaceae_UCG-002*, *Eubacterium_ruminantium_group*, and *Solobacterium* markedly increased at 75 d and decreased at 105 d. *Erysipelotrichaceae_UCG_ 002* is a member of the *Erysipelotrichaceae* family in the *Firmicutes* phylum and was previously reported to be involved in VFA synthesis [59] and the promotion of cholesterol production and accumulation [60]. Our results indicate that *Rikenellaceae_RC9_gut_group* and *Erysipelotrichaceae_UCG_002* exhibited contrary trends in the three age periods. We hypothesized a close relationship between the two. The correlation analysis further proved a negative correlation between the two. *Rikenellaceae_RC9_gut_group* was also negatively correlated with *Acetitomaculum* and positively correlated with TVFA. The relative abundance of the dominant bacterial genus *Acetitomaculum* increased significantly as age increased, with the highest relative abundance noted at 105 d (37%). Koike [61] found that *Acetitomaculum* can produce propionate from monosaccharides, which contributes to the synthesis of more propionate when solid diets are consumed. According to the correlation analysis, there is a significant positive correlation between *Acetitomaculum* and propionate and TVFA. This also further explains the higher propionate concentration in the rumen after weaning compared with that at pre-weaning. Meanwhile, this dominant genus interacted with several genera, and thus, it a promising target bacterium for regulating rumen function in calves. However, we found that the abundance of *Acetitomaculum* in this study was significantly higher than has been reported [62], which should be further investigated. *Eubacterium* is a crucial intestinal bacterium found in the colon of healthy humans. This bacteria is one of the core genera of the human intestinal microbiota and exhibits extensive colonization of the intestine, oral cavity, and other body parts of the majority of the population. *Eubacterium* has a critical role in the body, which includes nutrient metabolism and maintenance of intestinal homeostasis [63]. The *Eubacterium_ruminantium_group* can produce SCFA, and SCFAs are critical for energy balance, colonic motility, immune control, and suppression of intestinal inflammation [63]. The *Eubacterium_ruminantium_group* was positively correlated with TVFA in this experiment, and because weaning produces stress in calves, microorganisms adapt to this change with the conversion of diets and are more active at 75 d. The bacterial function prediction regarding enzyme-related genes also proved that weaning affected calves, especially in terms of the phosphoglucomutase (EC 2.7.11) gene that was more abundant at 75 d than at 45 d. It is a key enzyme in the glycolytic pathway, as the organism needs more energy probably because of weaning stress, and hence, it can produce more enzymes to further generate propionate, thereby providing energy for the organism. The variation in propionic acid concentration also indicates this. A recent study reported that bacterial-driven production of propionic acid and total VFA is strongly correlated with preweaning blood glucose and insulin, which are positively correlated with preweaning ADG [64]. Thus, we hypothesize that these rumen bacteria are essential in regulating the host phenotype. Overall, age change and diet transition are closely related. Interestingly, differences in rumen bacteria were noted even when the same diets were fed at 75 and 105 d. The changes in the bacterial composition may be due to age-related physiological changes.

Rumen fungi have pseudoroots and can generate highly active cellulases for degrading plant-based cell walls. They degrade and utilize plant-based diets to maintain the metabolic activities of the organism by collaborating with bacteria and protozoa in the rumen [65]. The study results unveiled that at the fungal phylum level, the dominant genera in the three age periods were *Ascomycota* and *Basidiomycota*. However, *Neocallimasatigomycota* has been reported to be the dominant fungi, which differs from this study, and we suspect that it may be due to differences in geographic environments or kit isolation methods. *Ascomycota* is a larger group of fungal microbes that are mainly involved in organic matter degradation in the nutrient cycle, such as that of lignin and keratin. *Ascomycota* is the dominant phylum of rumen fungi [66]; this is consistent with our findings. *Basidiomycota* is known to degrade mainly lignocellulose [67]. In our study, the relative abundance of *Basidiomycota* decreased at 75 d and increased at 105 d. This is possibly caused by diet transformation after weaning, which leads to changes in their abundance. With the increased intake of roughage, abundance increases and degradation is enhanced. Among the bacteria differing at the genus level, *Aspergillus* and *Xeromyces* had the highest abundance at 75 d, and they were positively correlated, whereas *Trichomonascus*, *Phialosimplex*, *Talaromyces*, and *Wickerhamomyces* were more abundant in the 105 d. *Aspergillus* is the most extensively investigated filamentous fungus, and it can produce ferulic acid esterase that partially degrades aromatic plant tissues, breaks covalent bonds, and collaborates with other enzymes to degrade lignin [68,69]. The relative abundance of *Trichomonascus* and *Talaromyces* increased with age, and *Aspergillus* and *Trichomonascus* exhibited a remarkable positive correlation with TVFA. Unfortunately, investigations on rumen fungi and their functions are limited. Knowledge about these rumen fungi and their functions need further in-depth studies. Most genes in the function prediction of fungal-related enzymes are related to carbohydrate and protein metabolism, which also demonstrates the critical contribution of fungi to diet digestion in the rumen.

## 5. Conclusions

This study on the rumen microbiota of calves revealed that rumen development gradually matures with age, and bacterial and fungal establishment gradually stabilizes. The three age periods were compared with each other. The rumen propionate concentration was significantly different. Weaning with diet transition significantly decreases rumen diversity in the short term and cause corresponding changes in the rumen microbiota composition. The production of propionic acid and total VFA may affect blood glucose and insulin, which in turn may have an effect on production performance, but further studies are needed to verify this.

In bacteria, *Bacteroides*, *Actinobacteriota* and *Firmicutes* were the core phylum for all age periods. At the genus level, *Ruminococcus*, *NK4A214_group*, *Sharpea*, *Rikenellaceae_RC9_gut_group*, and *norank_f__Butyricicoccaceae* were remarkably abundant at pre-weaning, and at 75 d, the relative abundance of *Erysipelotrichaceae_ UCG-002*, *Eubacterium_ruminantium_group*, and *Solobacterium* significantly increased. The relative abundance of *Acetitomaculum* increased as the age increased, with the greatest abundance noted at 105 d. In fungi, the dominant phyla were *Ascomycota* and *Basidiomycota*, and at the genus level, *Aspergillus* and *Xeromyces* were the most abundant at 75 d. *Trichomonascus, Phialosimplex*, and *Talaromyces* were significantly enriched at 105 d. However, the low abundance of *Neocallimastigomycota* was not detected throughout the study, which is worthy of further investigation. Moreover, correlations were observed between age-related abundances of specific genera and microbiota functions and rumen fermentation parameters.

This experiment expanded our understanding of the dynamics of microbiota in calves. The results revealed that the rumen microbiota structure can be changed by feeding of diets with different structures according to the specific physiological period of calves, and it may be an effective method to regulate rumen fermentation. It improves rumen health and performance. However, we need to further determine the functional characteristics of microbiota and interactions with hosts to identify the age-dependent patterns of microbial changes in the rumen.

## Figures and Tables

**Table 1:** Nutritional levels of diets.

Items	Nutrient Levels (DM Basis%)			
	CP	EE	NDF	ADF
Milk	3.5	3.2	-	-
Oaten Hay	9.35	2.44	53.41	28.6
Starter	22.2	2.8	12.2	6.1

(CP: Crude protein, EE: Ether extract, NDF: Neutral detergent fiber, ADF: Acid detergent fiber).

**Table 2:** The diet structure and the amount of intake of calves at different d of age.

Age	Diets	Total Dry Matter Intake (kg/day)	Nutrient Intake (g/kg DMI)			
			CP	EE	NDF	ADF
14–30 d	Milk + Starter	0.90 ± 0.04 ^e^	65.74 ± 6.60 ^d^	31.34 ± 0.14 ^b^	20.06 ± 4.30 ^f^	10.03 ± 2.15 ^d^
31–45 d	Milk + Starter + Hay	1.04 ± 0.05 ^e^	77.17 ± 7.15 ^c^	30.64 ± 0.13 ^c^	62.91 ± 2.92 ^e^	29.85 ± 8.14 ^c^
46–67 d	Milk + Starter + Hay	1.30 ± 0.11 ^d^	121.83 ± 8.79 ^b^	29.62 ± 0.14 ^d^	97.83 ± 2.12 ^d^	46.73 ± 10.46 ^b^
68–75 d	Starter + Hay	1.55 ± 0.15 ^c^	213.90 ± 0.86 ^a^	34.24 ± 0.66 ^a^	131.4 ± 5.21 ^c^	82.01 ± 13.24 ^a^
76–90 d	Starter + Hay	2.36 ± 0.26 ^b^	211.83 ± 1.11 ^a^	28.01 ± 0.78 ^e^	154.6 ± 3.56 ^b^	75.26 ± 8.81 ^a^
91–105 d	Starter + Hay	3.00 ± 0.17 ^a^	210.13 ± 0.68 ^a^	27.67 ± 0.02 ^e^	160.1 ± 2.19 ^a^	78.15 ± 10.02 ^a^
	*p* value	<0.001	<0.001	<0.001	<0.001	<0.001

Different letters represent significant differences, *p* < 0.05; same letters represent insignificant differences, *p* > 0.05.

## Data Availability

The datasets analyzed are not publicly available due to ownership by the funding partners, but are available from the corresponding author on reasonable request.

## Supplementary Materials

The following supporting information can be downloaded at: https://www.mdpi.com/article/10.3390/microorganisms12010012/s1, Table S1: Nutritional composition and nutrient levels of starter diets.
