# Supplementary material for: Effect of Dietary and Age Changes on Ruminal Microbial Diversity in Holstein Calves
Source: Microorganisms. 2023 Dec 20;12(1):12. doi: 10.3390/microorganisms12010012 (PMC10818949; doi:10.3390/microorganisms12010012)
Supplement: Supplementary file 1 [file microorganisms-12-00012-s001.zip › microorganisms-2724987-supplementary.pdf]

Table S1: Nutritional composition and nutrient levels of starter diets

Items	Proportions(%)
Ingredients	
Corn	45.7
Extruded soy	7.9
Soybean meal	28.5
Wheat bran	3.5
NaCl	0.5
Limestone	2.0
CaHPO4	0.6
Sugar beet pulp	7.3
Corn DDGS	3.0
Premix1	1.0
Total	100.0
Nutrient levels	
CP	22.2
EE	2.8
NDF	12.2
ADF	6.1
Ash	7.2
Ca	1.2
P	0.6
